# The Influence of Endodontic Lesions on The Clinical Evolution of Odontogenic Sinusitis—A Cohort Study

**DOI:** 10.3390/jcm12031103

**Published:** 2023-01-31

**Authors:** Marta Aleksandra Kwiatkowska, Kornel Szczygielski, Agnieszka Brociek-Piłczyńska, Aldona Chloupek, Dariusz Jurkiewicz

**Affiliations:** Department of Otolaryngology with Division of Cranio-Maxillo-Facial Surgery, Military Institute of Medicine—National Research Institute, Szaserów 128, 04-142 Warsaw, Poland

**Keywords:** chronic rhinosinusitis, odontogenic sinusitis, periapical lesion, apical periodontitis, root canal treatment, endodontic treatment

## Abstract

Endodontic disease with formation of periapical lesions (PALs) is one of the most common causes of chronic odontogenic sinusitis (ODS). It requires close collaboration between otolaryngologists and dentists, but the best sequence of management is still unknown. The aim of the present study is to clarify how radiological characteristics of teeth with PALs and previous root-canal treatment (RCT) influence the clinical evolution of the disease and to define the predictive value of its radiological and endoscopic features in determining the need for further surgical intervention. A total of 68 symptomatic patients with ODS with PALs were included in the study. The evaluation was performed by an otolaryngologist and a dentist based on a medical interview, nasal endoscopy, cold pulp testing and tomography images. Patients were prospectively followed for at least 12 months, during which nasal steroids, saline irrigations and RCT were administered. The criteria of disease improvement were: decrease of symptoms, healed sinonasal mucosa in endoscopy and radiological resolution of periapical radiolucency and sinus inflammation. Results showed that 9 (13%) patients improved after conservative treatment and 59 (87%) required further surgical intervention. Patients who improved after medical treatment and RCT were younger (*p* = 0.043) and had a greater distance from the top of the periapical lesion to the maxillary sinus’ floor (*p* = 0.003). When expansion of PALs and bone destruction toward the maxillary sinus was observed on radiological imaging (*p* = 0.041), and when more than one tooth root was affected (*p* = 0.004), patients were more likely to require surgical intervention. In conclusion, the more roots that are affected and the closer the top of the PAL is to the maxillary sinus’ floor, the greater the possibility of medical treatment and RCT failure. When the bone destruction extends into the maxillary sinus, patients eventually require both tooth extraction and FESS in order to resolve ODS completely.

## 1. Introduction

Odontogenic sinusitis (ODS) is a primarily maxillary sinusitis that may also affect other sinuses. It is caused by the spread of bacteria, toxins and proinflammatory cytokines through the thin maxillary bone from the adjacent dental pathology [1,2].

The prevalence of ODS used to be highly underestimated, but during the last decade, several reports documented the suspicion of an odontogenic source of maxillary sinusitis in up to 70% of unilateral cases [3,4].

Dental caries and periapical pathology are one of the most common causes of ODS [4,5] and represent a subtype of the disease. Consequently, its management differs from sinusitis caused by iatrogenic dental procedures or trauma [1,6]. Although many authors undoubtedly distinguish ODS as a condition with distinct pathophysiology and treatment outcome from ‘rhinogenic’ sinusitis, scientific literature has a substantial lack of observational and prospective studies regarding individual causative dental diseases. Various published studies are based only on radiological findings [2,5,7], whereas all types of sinusitis, by definition, require sinonasal symptoms to be present [5,7]. Diagnosis of ODS should be based on clinical and radiological examination of the involved teeth and sinuses and requires thorough collaboration between otolaryngologists and dentists [1,7,8].

What makes the diagnosis of odontogenic sinusitis even more complicated is that some patients may already have a primary rhinogenic disease before the odontogenic involvement occurs. There has been a great deal of debate in the dental and medical literature regarding the proper treatment of this condition, but to date neither rhinological [9] nor dental [7] guidelines provide the exact sequence of management.

If patients suffering from ODS with PALs are referred to an otolaryngologist, first-line medical therapy is usually applied. In order to treat the mucosal edema and reestablish the patency of ostiomeatal unit, nasal steroids and saline irrigations are often recommended [9].

Concerning treatment options for the causative tooth, proper root-canal treatment (RCT), apicoectomy or a combination of these methods usually results in satisfactory healing of PALs [3,10].

It is proven that dental management might be sufficient to eradicate the source of infection, but it cannot restore sinonasal mucociliary clearance [11]. If ODS does not resolve after conservative treatment, patients must consider tooth extraction, functional endoscopic sinus surgery (FESS), or both [12].

Another problem is the completeness and evaluation of RCT. Efficacy data for dental treatment is limited [1]. Teeth that only have filling material in the pulp chamber, or those with unfilled or missed root canals are still a potential source of infection [13]. In the literature, extraction of the causative tooth is the only primary dental treatment with published success rates [12].

Choosing the optimal sequence of treatment is still difficult because of the low publication volume of prospective studies and logistical challenges of coordinating multidisciplinary care between otolaryngologists and dental specialists [2,5,12]. Usually, the medical literature focuses either on laryngological treatment of ODS and its results or the resolution of periapical lesions after endodontic treatment.

Several studies have demonstrated convincing results after surgical treatment, but the ideal sequence of conservative and invasive procedures remains unclear [1,4,6,7,8,10,12]. Moreover, recently published consensus statements did not provide the exact radiological or endoscopic characteristics that would guide clinicians to qualify the patient for surgery.

The rationale of this study was to define the predictive value of radiological and clinical features of odontogenic sinusitis of endodontic origin in order to provide the most exact and effective treatment and spare the patient unnecessary and costly procedures. Working in close collaboration with otolaryngologists, maxillo-facial surgeons and dental specialists, we aimed to observe and comprehend the effectiveness of first-line treatment from a multidisciplinary perspective.

The main aim of the current study was to clarify how the endoscopic score of nasal cavities, radiological characteristics of teeth with periapical lesions and previous root-canal treatment influence the clinical evolution of odontogenic sinusitis of endodontic origin and the possibility of disease resolution after conservative treatment. The secondary objective was to define the predictive value of periapical lesion’s radiological features based on the Estrela scale to determine the need for further surgical intervention.

## 2. Materials and Methods

### 2.1. Study Design

A cohort study was designed to assess the objectives. The conservative treatment of odontogenic sinusitis was implemented, and patients were prospectively followed for at least 12 months. In order to confirm the diagnosis of ODS with PALs, patients were evaluated by both an otolaryngologist and a dental specialist. Laryngological examination consisted of a medical interview, endoscopic examination of nasal cavities and CT and/or CBCT imaging of the sinuses and adjacent maxillary dentition.

Medical therapy for chronic rhinosinusitis with saline irrigations and nasal steroids (100 µg of mometasone furoate to each nostril once daily) were administered for at least 3 months. In cases of previous RCT of tooth with persistent PAL, the treatment’s quality was established together with the dental specialist. Re-endodontic therapy was administered if needed. If the causative tooth was not treated, endodontic consultation and treatment were advised. The criteria of disease improvement were a decrease of endoscopic score and total resolution of periapical radiolucency and sinus’ mucosal inflammation on CT or CBCT scan.

Patients who failed medical treatment and presented with persistent PALs or refused to undergo RCT because of economic reasons were consulted one more time and offered surgical treatment (either FESS and/or tooth extraction) based on a collaborative decision between the patient and the dental care provider.

### 2.2. Setting

The cohort was recruited from inpatient and outpatient units of the Department of Otolaryngology with the Division of Cranio-Maxillo-Facial Surgery in Military Institute of Medicine, Warsaw, Poland.

### 2.3. Participants

Adult patients who presented with symptoms of homolateral chronic rhinosinusitis [9] and a periapical lesion around maxillary molars or premolars were included in the study. Patients with oroantral fistula after previous tooth extraction or other surgical interventions in oral and/or sinonasal cavities, patients with more than one tooth with PAL on the same side and patients with radiological features of OS with PALs but who were asymptomatic were excluded from the study.

### 2.4. Data Collection and Measurements

The following demographic and clinical data were gathered: age, gender, number of tooth and roots affected by PAL, extension of sinusitis and ostiomeatal complex obstruction. Sinus endoscopy was scored according to a modified Lund–Kennedy Scale, which grades polyposis, discharge and edema on an ordinal scale from 0–2 for each side. Higher scores indicate worse observed disease. A modified scale was implemented because it eliminates the scarring and crusting items and better correlates with the symptoms in patients without previous sinus surgery.

Dental evaluation of periapical tissues was performed by the same dental specialist and consisted of percussion, palpation, mobility tests, cold pulp testing and analysis of CT and/or CBCT scans. If the affected tooth with a periapical lesion had undergone previous endodontic treatment, its effectiveness was established considering untreated or sub-optimally filled canals, inadequate core restorations or leaking canal restorations [6,14,15].

Radiological analysis consisted of multiplanar reconstruction of CT and/or CBCT scans involving paranasal sinuses and maxillary dentition. Mucosal changes exceeding 2 mm within the ostiomeatal complex and/or sinuses were considered pathological [9]. The PALs were scored according to the Estrela scale [14], which is a 6-point scoring system, used with 3 additional variables: E-bone expansion and D-bone destruction of cortical bone. The incidence of destruction towards the maxillary sinus and absence of cortical bone over the periapical lesion was also noted. To distinguish destruction of bone towards maxillary sinus from buccal direction the first incidence was marked as M. Distance from the top of PAL to the maxillary sinus’s floor was measured separately in each plane. The mean value of the three measurements was then extracted.

When more than one root was involved, the measurements were completed separately for each root, and the root that had the apical lesion with the largest dimension was analyzed. If the radiolucency involved the furcation, adjacent roots were considered as affected with PAL.

**Figure 1:**
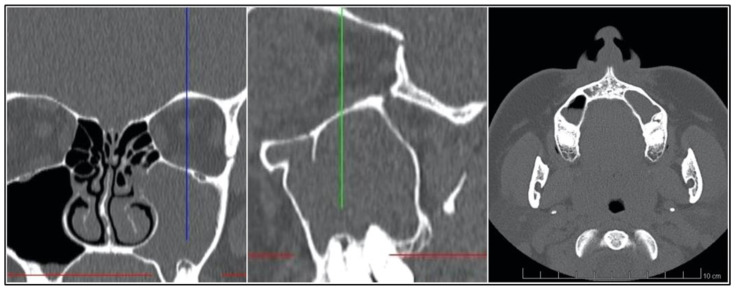
The example of CT scans of odontogenic maxillary sinusitis with a periapical lesion affecting mesio- and distobuccal root of first maxillary molar; from left to right: coronal, sagittal and axial view. Vertical and horizontal lines point the PAL.

The example of the PAL around the first maxillary molar affecting mesio- and distobuccal root with odontogenic sinusitis is presented on Figure 1.

### 2.5. Study Size

At first, 73 patients fulfilled the inclusion criteria, but 5 of them did not complete the appointed control visits. Consequently, 68 patients were enrolled in the study.

### 2.6. Statistical Analysis

Statistical analysis was performed using Statistica software version 13.1 (©StatSoft Polska). Qualitative variables were presented as a percentage distribution; for quantitative variables, the mean, standard deviation (±SD), median and range were given. The Kruskal–Wallis test was used to assess all independent, no-parametric variables for significant differences. The endoscopic score of nasal cavities, radiological characteristics of teeth with periapical lesions and previous root-canal treatment influenced the clinical evolution and possibility of disease resolution after conservative treatment were considered. The Chi-squared test was used to determine whether demographic variables influenced the need for surgical intervention. The level of statistical significance was set at 0.05. Statistically significant correlations were checked with the use of non-parametric Mann–Whitney U test as the collected data were ordinal, and the assumptions of the independent *t*-test were not met.

### 2.7. Statement of Ethics

This study has been carried out in accordance with “The Code of Ethics of the World Medical Association (Declaration of Helsinki)” for experiments involving humans.

It was approved by the Ethics Committee of the Military Institute of Medicine (protocol No 43/WIM/2019) and written informed consent was obtained by each participant.

## 3. Results

A total of 68 patients, with 33 women (48.5%) and 35 men (51.5%), were enrolled to the study. Mean age of the group was 49.3 ± 13.6 years, median: 49 years (range from 22.6 to 78.9 years).

Of the 68 patients, 8 (13%) improved after conservative treatment and 60 (87%) required further surgical intervention. The demographic details are given in Table 1.

There was a statistically significant relationship between the failure of conservative treatment and the variables of the Estrela scale (*p* = 0.041) as well as with the distance from the top of the periapical lesion to the maxillary sinus’ floor (*p* = 0.003). Diameter of the periapical lesion did not affect significantly the need for surgical intervention (*p* = 0.605). In the conservative treatment group, the median score on the Estrella scale was 3.5 (range from 1 to 4), while in the surgical treatment group, the median value was 4.0, ranging from 3.0 to 5.0.

Comparisons of the modified Estrela scale variables and their combinations present as follows: in the group that improved after conservative treatment, only expansion (E) was much more common (85.7% vs. 36.7% in the surgical group). Various combinations of expansion and bone destruction appeared much more often in the surgical than conservative group (58.3% vs. 14.3% respectively). Detailed descriptive statistics of the reported parameters are given in the Table 2.

An important correlation was also found for the number and type of affected roots with periapical lesions and the possible need for surgical intervention (*p* = 0.004). The total number of tooth roots with periapical lesions in the surgical group was greater than in conservative group. When the PAL involved all roots, the conservative therapy was only sufficient to resolve the disease in one case. Buccal roots were more often affected than palatal ones in the presented study. Table 3 presents the exact values of the abovementioned comparisons between treatment groups.

No statistical significance was found for the type of causative tooth (molars or premolars) and its relation to the possibility of conservative treatment’s failure. Nevertheless, molars were affected much more often than premolars: 49 (72.1%) vs. 17 (25%) of all cases, respectively.

No statistically significant correlations were found when prospectively comparing the need for surgical intervention with either the patients who had previously undergone RCT with a good healing result (RCT complete) or the patients who had not received RCT at all during follow-up or had RCT that was considered incomplete. The number and percentage of patients in each treatment group and the quality of endodontic treatment are given in the Table 4.

Concerning the otolaryngological evaluation during endoscopy, an important correlation was found for discharge (*p* = 0.01). In 90% of cases, patients who eventually needed surgical intervention had visible secretions in the middle meatus. It was thick and purulent in half of the patients, and thin and mucous in the other half. Table 5 presents the rest of results along with their statistical significance.

In the group of patients who improved after conservative treatment, CT scans revealed at least sinus partial opacifications, but the ostiomeatal unit remained patent. Out of the 60 patients who needed further interventions, 47 (78.3%) presented the obstructed ostiomeatal unit. The obtained p value is <0.001, which indicates the existence of a relationship between the patency of the ostiomeatal unit and the need for surgical intervention.

## 4. Discussion

Endodontic disease with formation of periapical lesions is one of the most common causes of chronic odontogenic sinusitis [16,17,18,19,20], especially if the lesion lacks a definitive border or is in close proximity to the sinus [18,21,22,23].

Bacterial invasion spread along the tooth root, beyond the apical structure and into the surrounding periapical tissue [21]. At first, chronic inflammatory reactions of sinus mucosa are limited to the area directly above the periapical lesion [4]. However, a secondary rhinogenic infection may also become involved in the ongoing process and cause the subsequent ostial obstruction [2,11,21].

In our study, only symptomatic patients were included. Concerning radiological features of PALs, although their size showed no statistical significance in prediction of the need for surgical intervention, other variables of the Estrela scale could be suggestive in choosing the proper management. The presented study showed that if only the expansion of the periapical area is noted, the greater is the possibility of resolving the odontogenic sinusitis after conservative treatment. If there is destruction of alveolar bone in the direction of the maxillary sinus, there is a stronger possibility that patient will eventually require surgical intervention. The described variables of the Estrela scale resulted in be the most suggestive radiological parameter to consider while planning further treatment of ODS.

Although the type of affected teeth (premolars or molars) did not significantly influence the prospective outcome of treatment, it did show that when more roots were altered at the beginning, it was more likely that the conservative therapy would fail. The roots of the maxillary teeth may also protrude into the maxillary sinus, with a separation of thin layer of mucosa and/or bone (thinnest over the second maxillary molar) [19]. Various studies reported that first and second maxillary molar are more likely to develop periapical lesions and subsequent ODS [11,15,16,17,24].

In the current study, molars were affected with PALs more often than premolars, although without statistically significant impact on the possibility of conservative treatment’s failure. Gender was not significantly correlated with the conservative treatment failure, in contrast to the age. The median age of the 8 people that successfully healed with conservative treatment was significantly lower than that of the patients who required surgical treatment. A higher prevalence rate of sinus membrane thickening was found in older age groups in various retrospective studies, such as the one presented by Huang et al. Patients in the age group ≥ 60 years had a 4.35 times greater risk of sinus membrane thickening than the 35-year-old patients [25]. Elders’ teeth reflect the accumulation of oral disease and quality of dental care, they may have previously undergone RCT or retreatment, and they are more likely to loose teeth due to caries, periodontal or pulpal disease [25]. Studies also suggest that the calcification and morphologic changes that occur in root canals with age makes proper RCT increasingly difficult [26].

Previous studies from our database showed that when PAL had a larger diameter and was closer to the maxillary sinus floor, a greater extent of sinus opacification and a higher possibility of ostiomeatal complex obstruction was observed [20,27].

As reported by Souza-Nunes et al. the size of the periapical lesions and their proximity to the maxillary sinus floor had a high association with the appearance of sinus abnormalities [24]. In their study, significant associations were found for a lesion with a diameter greater than 5 mm, involvement of more than 1 tooth root and/or the furcation region and cortical bone destruction. In a retrospective radiological study by Nunes et al., there were no significant differences between teeth (premolars or molars) and the presence or absence of MS changes. Most of the sinus abnormalities were found when the distance between the periapical lesion edge and the MS was 0 and when the size of PAL was in the range of 4–8 mm [28], which corresponds with point 4 in the Estrela scale.

A negative correlation between the distance of the tooth root apex to the floor of the maxillary sinus and the thickening of the maxillary sinus mucosa was also stated by Kuligowski et al. [15]

However, in the study presented by Lu et al., the spatial relationship between MS and infected roots did not significantly affect the presence of sinus abnormalities [29]. The difficulty in comparing our results to existing literature is the limited availability of prospective studies that analyze more than radiological images. Therefore, it is complicated to judge whether “sinus abnormalities” could be interpreted as sinusitis, which is an entity diagnosed by its clinical presentation [5,9,10].

Evaluation of ODS of endodontic origin is also particularly challenging as formation of the PAL may not always be visible on CBCT scans [5]. However, if ODS patients had prior endodontic treatment, the clinical pulp test will have a negative result. That leaves radiographic images that show the presence of PAL as the only suggestion of the endodontic source of infection [5].

In our cohort, the rate of complete RCT identified after clinical and radiological examination performed by a dental specialist was quite low. Unsuccessful outcomes of RCT may be due to the persistent intra- or extra-radicular infection. It is also important to follow patients for at least a 12-month interval, when 88% of periapical lesions exhibit signs of healing [30]. According to Signoretti et al. the clinical success of an endodontic retreatment seems to be related to the management of the alterations in the natural course of the root canals caused by previous interventions [4,31]. The prevalence of tenacious apical periodontitis and other periradicular diseases can transcend 30% of the total root-filled teeth population [28]. When Czarnecka et al. analyzed patients with ODS, they diagnosed 48.9% teeth with improper RCT [17]. In material presented by Dobroś and Zarzecka, out of 13 causative teeth with PALs that were treated endodontically, only a single tooth had a properly reconstructed crown, and only one tooth had an adequately filled-in root canal [13]. On the other hand, Sato et al. analyzed patients with persistent OS with PAL after medical and endodontic therapy and observed that 97.6% of causative teeth were able to be preserved without dental retreatment. In two cases, extraction was necessary because of the tooth mobility [16].

In the literature, various radiological scales were proposed to evaluate the periapical tissue and RCT outcomes [14,28,32,33], but their clinical evaluation was usually not reported. Gudac et al., after 24 month of observation, stated that even the complex periapical and endodontic status scale [32] did not demonstrate satisfactory potential to predict a reliable treatment outcome for the teeth assigned to the moderate- and high-risk groups [32]. Nevertheless, none of the aforementioned scales simultaneously analyzed the paranasal sinus’ condition.

Concerning radiological features of sinuses, Souza–Nunes et al. [24] did not find any statistically significant impact of endodontically treated teeth on the severity of sinus mucosal thickening, which is also in accordance with the results of the current study.

One of the limitations of our study is that some patients had already received first-line root-canal treatment before being assigned to the study, and data regarding it’s exact technique were not available. At the same time, despite being informed about the root-canal treatment possibility which would have spared the causative tooth, economic reasons caused many patients to resign from the study and opt for an extraction. That may influence the overall outcome of conservative therapy in favor of surgical interventions and cause bias in the result’s interpretation.

Previously published studies showed that the extraction of highly damaged teeth usually reduces the inflammation of maxillary sinus [24,32]. Yilmaz reported a case of a periapical lesion with an oroantral fistula of 2 mm in diameter above the mesiobuccal root apex. Endodontic treatment was initiated, and a CT scan of the paranasal sinuses performed after 3 years revealed the resolution of sinus’ mucosal inflammation and formation of a new alveolar bone [21]. Even though our follow-up period was not equally long, when medical therapy and RCT were complete, we observed improvement in the overall score on endoscopic evaluation as well as a decrease in radiological sinus opacification and thickness of maxillary sinus’ mucosa, which was interpreted as healing of sinusitis.

Various authors suggested addressing the dental issue as a first line of therapy for ODS [7,11], while others propose FESS for intractable ODS caused by RCT and dental restoration, with subsequent close dental follow-up [4,16,33]. It is essential to emphasize the potential for the development of odontogenic sinusitis caused by the teeth with PAL.

PALs around molar or premolar tooth apexes with coexistence of unilateral sinus partial or total opacifications should always be evaluated by dental specialists and otolaryngologists. Meticulous clinical examination should involve nasal endoscopy and analysis of CT or CBCT images. Expansion of PALs with destruction of cortical bone, especially in the direction of the maxillary sinus and the presence of discharge on a nasal endoscopy, might suggest failure of conservative treatment and the need for further surgical interventions. A group of patients in this situation should be closely followed by otolaryngologists and dentists in order to choose the best way to further manage ODS of endodontic origin and avoid inadequate and often costly procedures.

Given the lack of complete prospective data that would distinguish the differences in clinical evolution in subtypes of odontogenic sinusitis in the literature, further prospective research is needed.

## 5. Limitations

There are some limitations to this study. First, the data was collected from a single institution. A multicenter prospective study would be beneficial to compare these findings. Second, only symptomatic patients with visible PALs on radiographic images were enrolled. This might underestimate the existence of endodontic-related odontogenic sinusitis even in a single-center cohort, as apical periodontitis does not always cause symptoms or result in visible PAL around the infected tooth root. Moreover, first-line dental examination and root canal treatment were not performed by the same doctor, so data on the exact procedures were not available. Only the completeness and clinical result of RCT was evaluated in collaboration with endodontists and maxillo-facial surgeons. State-provided dental treatment in Poland also does not cover the endodontics. Economic reasons cause many patients to resign from conservative therapy and opt for tooth extraction. That may influence the overall outcome of conservative therapy in favor of surgical interventions.

## 6. Conclusions

- Nasal steroids and root-canal treatment rarely resolve OS with PALs completely.
- The closer the top of the periapical lesion is in relation to the maxillary sinus’ floor, and the more roots are affected with PAL, the greater the possibility that conservative treatment will not be sufficient.
- In our cohort, no statistically significant correlations were found for the need of surgical intervention among patients who had previously undergone RCT with a good healing result and those who either had not received RCT at all during follow-up or the RCT was considered incomplete.
- Expansion of PALs expansion with destruction of cortical bone, especially in the direction of the maxillary sinus, as well as the presence of discharge on nasal endoscopy is suggestive of conservative treatment failure and the need for further surgical interventions. Patients in this situation should be closely followed by otolaryngologists and dentists in order to implement and perform more invasive procedures.

## Figures and Tables

**Table 1:** Descriptive statistics of demographic in the study group; Median age is presented in years with range given in the brackets.

	Conservative Treatment	Surgical Treatment	*p*-Value
Women	5 (62.5%)	28 (46.7%)	*p* = 0.385
Men	3 (37.5%)	32 (53.3%)	
Age median (range)	43 years (23–67)	50 years (22–79)	*p* = 0.043

**Table 2:** Estrela scale variables (number and percentage) of periapical lesions and their correlation with the necessity of surgical intervention. Bone expansion–E, bone destruction–D, bone destruction towards the maxillary sinus–M or none–0 and their combinations.

*p* = 0.041						
Estrela’s Scale Variables Type of Treatment	0	E	ED	EDM	EM	D
Conservative	0 (0%)	6 (75%)	1 (12.5%)	1 (12.5%)	0 (0%)	0 (0%)
Surgical	3 (5%)	22 (36.7%)	10 (16.7%)	22 (36.7%)	2 (3.3%)	1 (1.7%)

**Table 3:** Descriptive statistics (total number and percentage) of type of affected roots with periapical lesions in each of the subsequent treatment groups of patients; *p* = 0.004.

*p* = 0.004	Affected Roots						Affected Teeth	
Type of Treatment	All Roots	Distobuccal	Mesiobuccal	Disto- and Mesiobuccal	Palatal	Distobuccal and Palatal	Molars	Premolars
Conservative	1 (12.5%)	1 (12.5%)	2 (25%)	1 (12.5%)	3 (37.5%)	0 (0%)	5 (62.5%)	3 (37.5%)
Surgical	18 (30.5%)	2 (3.3%)	5 (8.5%)	19 (32.2%)	8 (13.6%)	7 (11.9%)	44 (74.6%)	15 (25.4%)

**Table 4:** The amount and percentage of patients in each treatments group and the quality of root-canal treatment.

*p* = 0.10			
Type of Final Treatment	RCT Incomplete	RCT Complete	Lack of RCT
Conservative	3 (37.5%)	5 (62.5%)	0 (0%)
Surgical	26 (43.3%)	11 (18.3%)	23 (38.3%)

**Table 5:** Descriptive statistics (number and percentage) and p-values of endoscopic presentation in each treatment group, based on the modified Lund–Mackay scale.

Lund–Kennedy Scale	Type of Treatment	
	Conservative	Surgical
Edema	*p* = 0.113	
0	3 (37.5%)	14 (41.3%)
1	5 (62.5%)	46 (76.7%)
Discharge	*p* = 0.010	
0	3 (37.5%)	6 (10%)
1	5 (62.5%)	27 (45%)
2	0 (0%)	27 (45%)
Polyps	*p* = 0.735	
0	8 (100%)	53 (88.3%)
1	0 (0%)	5 (8.3%)
2	0 (0%)	2 (3.3%)

## Data Availability

The data that support the findings of this study are not publicly available as they contain information that could compromise the privacy of research participants, but are available from the corresponding author M.K.
